# *attract:* A Method for Identifying Core Pathways That Define Cellular Phenotypes

**DOI:** 10.1371/journal.pone.0025445

**Published:** 2011-10-14

**Authors:** Jessica C. Mar, Nicholas A. Matigian, John Quackenbush, Christine A. Wells

**Affiliations:** 1 Department of Biostatistics, Harvard School of Public Health, Boston, Massachusetts, United States of America; 2 Department of Biostatistics and Computational Biology, Dana-Farber Cancer Institute, Boston, Massachusetts, United States of America; 3 National Centre for Adult Stem Cell Research, Eskitis Institute for Cell and Molecular Therapies, Griffith University, Brisbane, Queensland, Australia; 4 Department of Cancer Biology, Dana-Farber Cancer Institute, Boston, Massachusetts, United States of America; 5 Australian Institute of Bioengineering and Nanotechnology, University of Queensland, Brisbane, Queensland, Australia; Semmelweis University, Hungary

## Abstract

*attract* is a knowledge-driven analytical approach for identifying and annotating the gene-sets that best discriminate between cell phenotypes. *attract* finds distinguishing patterns within pathways, decomposes pathways into meta-genes representative of these patterns, and then generates synexpression groups of highly correlated genes from the entire transcriptome dataset. *attract* can be applied to a wide range of biological systems and is freely available as a Bioconductor package and has been incorporated into the MeV software system.

## Introduction

The molecular networks that define the phenotype of a cell can be captured through global gene expression profiling, however many cellular functions are shared between different cell types, and identifying those features that best discriminate between phenotypes remains a challenge. There are a number of reasons for this, including a lack of methodologies that simultaneously compare between multiple phenotypes and the general reliance on ranked gene lists that are associated with phenotypes in a post-hoc manner.

The basis for most expression-based analyses is the search for genes that exhibit patterns of differential expression between phenotypic or experimental groups, followed by meta-analysis to identify potential functional interpretations of the resulting gene lists. This is true for most approaches focused on identifying co-expression networks from microarray data [1], [2], [3]. A general workflow takes the initial significant gene list and reduces it based on a post-hoc application of knowledge about the potential functional roles that the selected genes play. While this is a useful way to annotate large datasets, it often restricts subsequent analyses to well-annotated genes.

Here we describe *attract*, an approach that leverages both existing pathway databases and the differences in the expression of the genes in those pathways between multiple cell types. *attract* expands these inferences by identifying new co-ordinately-regulated gene sets that are relevant to the mechanisms underlying the phenotypic differences that define specific cell types.

We apply *attract* to the four most phenotypically diverse cell types analyzed by Müller et al. (NCBI GEO accession number GSE11508). Müller and colleagues generated a library of stem and progenitor cells and used gene expression to define groups based on their degree of pluripotency [1]. Using an unsupervised machine learning method, they found that the human undifferentiated pluripotent stem cell lines (PSCs) were highly correlated in their expression profiles, whereas other lines, and in particular brain-derived neural stem cell lines, were more heterogeneous and apparently similar to other stem cell types. Having defined pluripotency classes, they used MATISSE [2] to construct a transcription factor network centered on the pluripotency factors Oct3/4 and Nanog, and presented this PSC-derived network, the “PluriNet,” as a resource for characterizing stem cell lines.

PluriNet was an important step forward in applying gene expression-based phenotypes to stem cell classification; however the broader implications of this result have only received limited attention. It is not clear how generalizable the PluriNet is to other stem cell models, nor what biological pathways or interacting networks are differentially activated to define such diverse cell types. Our analysis focused on embryonic stem cells (ESCs H9, Miz4, Miz5, Miz6), neural stem cells (Nlin9, Nlin10), neural progenitors (NLin15) and testicular teratocarcinoma (NTera2); these types were derived from a range of different tissue sources, were well replicated, and spanned the spectrum of pluripotent abilities. We demonstrate the power of *attract* to find meaningful, discriminatory gene expression modules in this stem cell model system.

## Analysis

### The “attract” method

**Figure 1:**
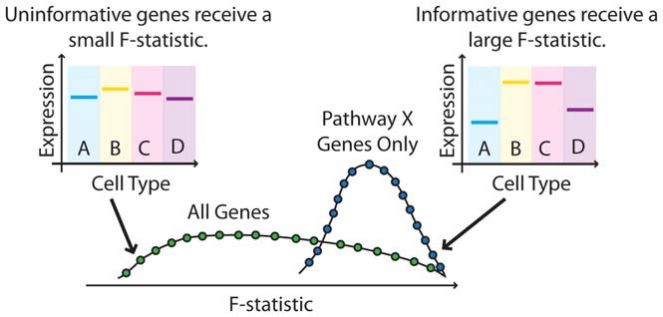
Schematic overview of *attract*.

*attract* is a modular process that consists of the first three steps summarized in Figure 1, and is essentially the inverse of more traditional gene expression analysis approaches. First we test the 'foundation knowledge sets' to identify those well-annotated gene-sets (for example, KEGG pathways) that best discriminate between cell-phenotypes. Next we identify the 'discriminating profiles' 'by decomposing each gene-set into profiles which summarize the differential expression across the sample groups. Finally, we build 'correlated gene sets' which extend the analysis to the entire expression dataset by identifying those genes which are highly correlated with these discriminating patterns. In this way, we start from a strong knowledge-base position which permits hypothesis driven exploration of the data as a whole. The end point of this pipeline is a gene-discovery set with function inferred by virtue of co-regulation with known biological processes.

**Figure 2:**
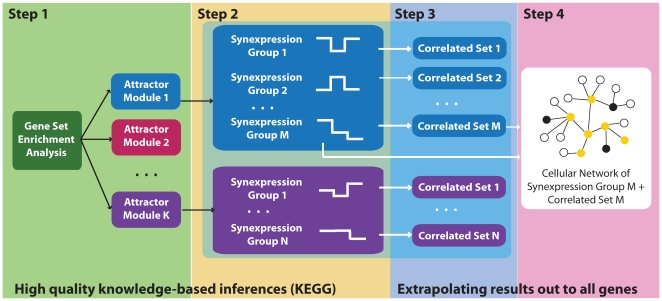
The ANOVA-based step of *attract*—a novel gene set enrichment implementation. Each gene is assigned an F-statistic where consistent cell-type specific changes are up-weighted. Pathways that have distributions of F-statistics distinct from the global distribution are flagged as significantly enriched for cell-type specific expression changes.

To test pathway-level data we developed GSEA-ANOVA, an analysis of variance-based implementation of a gene set enrichment algorithm (Figure 2). Unlike other GSEA implementations which only allow for two-class comparisons, this ANOVA-based approach tests for differences between multiple classes. Under GSEA-ANOVA, we fit an ANOVA model to each gene where a gene's expression is modeled by a single factor representing the cell types as distinct levels of this class. For instance, for gene *i* and its corresponding expression value in each replicate sample *j  =  1, …, r_k_* for each cell type *k  =  1, …, K,* we fit the following fixed effects model: 
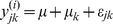
(1)where *µ* reflects the overall mean, *µ_k_* represents the effect of cell type group *k* on the gene's expression, and *ε_jk_* is the random normal residual error term.

Under the null hypothesis *H_0_*: *µ_1_  = µ_2_  = …  =  µ_K_,* the assumption is that all *K* cell type means are equivalent, or in other words, that there are no expression changes associated with cell type groups. The mean expression for cell type *k* is given by: 
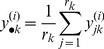
(2)


From the ANOVA model, we compute the *F*-statistic for gene *i*:
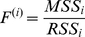
(3)where *MSS_i_* represents the mean treatment sum of squares, and captures the amount of variation due to the cell type group-specific effects: 
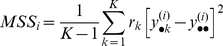
(4)and *RSS_i_* represents the residual sum of squares:
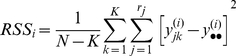
(5)where N is the total number of samples, and the overall mean is given by:
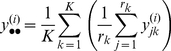
(6)


The *F*-statistic captures the strength of association observed in a gene's expression over the different cell types. Large values of the *F*-statistic indicate a strong association whereas a small *F*-statistic suggests that the gene demonstrates minimal cell type-specific expression changes.

The ANOVA model and the corresponding F-statistic it produces, gives us a way to gauge which genes are informative for a particular set of cell types. Our main interest however lies in understanding which pathways collectively consist of genes that together inform us of enrichment for a celltype through consistent cell type-specific changes. In the current implementation, we map genes on the array to KEGG pathways [3] although other pathway databases could be substituted. Since large F-statistics are indicative of strong cell type-specific changes, a pathway whose distribution of F-statistics is skewed towards larger values represents an enrichment in expression changes that inform us of cell type. To test this relationship more formally, we appeal to a two sample T-test to compare the distribution of log_2_-transformed F-statistics from all pathway members to the global distribution of log_2_-transformed F-statistics from all genes with a pathway annotation. The log-transformation is necessary to satisfy the normality assumption underlying the T-test and a Welch modification is used to protect against instances where the variances are unequal between the two groups under comparison.

For pathway *P* consisting of *g_p_* genes, the *T*-statistic takes the following form: 
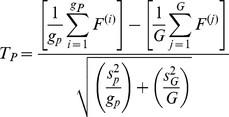
(7)where *G* represents the total number of genes with a pathway annotation and the sample variances 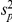 and 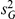 are defined as: 
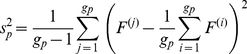
(8)

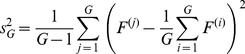
(9)and the degrees of freedom are specified by the Welch-Satterwhaite equation: 
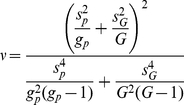
(10)


While other tests could be substituted instead to test for differences between the pathway distribution and the global distribution of *F*-statistics, it has been recently suggested that the T-test, as opposed to other tests such as the Kolmogorov-Smirnov test which is known for its lack of sensitivity, is simpler and more practical solution [4]. Given the volume of pathways available in resources like KEGG, we must address the multiple-testing issue and this is accounted for by adjusting the resulting P-values using a Benjamini-Hochberg FDR-based method [5]. Pathways with distributions significantly different from the global distribution are those best able to discriminate between the cell types of interest.

The second step in *attract* summarizes each significant, discriminative pathway into subsets of genes in which members have very similar patterns of expression. We refer to these subsets as “synexpression” groups, a term originally used by Niehrs and Pollett [6] to describe coordinately expressed genes with inferred co-regulation. Synexpression groups are obtained by by decomposing each significant pathway into correlated subsets using hierarchical clustering based on a Pearson correlation coefficient distance measure. The optimal number of synexpression groups was determined using an informativeness metric, a method which assesses the maximum number of clusters that provide consistent expression profiles that provide the most amount of information regarding cell type or sample-type specific changes [7].

The third step in *attract* is a discovery step in which we extend each synexpression group to include all genes that exhibit highly correlated expression patterns. For each synexpression group, correlation coefficients are computed between genes annotated to the synexpression group and the set of unannotated genes. Functional relationships are inferred for those unannoted genes that meet a user-specified cutoff for the correlation coefficient (by default, 0.85). Most sources of annotation, including KEGG, still represent a minority of genes and we have found great value in using the data to extend putative functional annotation of gene sets. This empirical knowledge-based approach allows functions and pathway associations to be inferred for genes which have no annotation.

Our method has been implemented in an R package attract and is available from Bioconductor. The data set and code used in these analyses, including attract, can be downloaded from http://compbio.dfci.harvard.edu/pubs/attractsupplement.zip.

## Results

### attract identifies the major biological themes in the Müller dataset

The implementation of attract is demonstrated on the Müller Plurinet dataset (NCBI GEO (accession number GSE11508 [1]), to identify the pathways that best describe four exemplar cell types. A subset of cell types representing embryonic stem cells (ESCs), neural stem cells, neural progenitor cells, and teratocarcinoma-differentiated cells (teratocarcinomas) were extracted analysis restricted to the same platform: in this case, the Illumina WG-6 BeadChip array. The resulting data set had 68 samples: 12 ESCs, 31 neural stem cells, 8 neural progenitors, and 17 teratocarcinomas. We applied a quality filter to the gene expression data where a probe was retained if it had a 0.99 detection score in 75% of samples in at least one of the four cell types.

The GSEA-ANOVA is based entirely around an ANOVA framework, and it is worth pointing out that the practical rules of good experimental design associated with standard linear models, are also relevant to the application of *attract* to gene expression data sets. Namely, replicates go a long way in improving the fit, accuracy and stability of the linear model, and there should be at least three replicates (ideally, many more) for each cell type or sample group. Cell type group sizes should also be reasonably consistent, and within the same order of magnitude at least.

Using the Bioconductor annotation package illuminaHumanv1.db version 1.6.0 there are 47, 289 probes on the Illumina WG-6 BeadChip, of which 5, 668 (12.0%) are assigned to one or more KEGG pathways. When applied to the embryonic stem cell, neural stem cell, neural progenitor, and teratocarcinoma cell types in the Müller dataset, *attract* identified eleven significantly enriched pathways (P-value<0.05, Table 1). These can be broadly classified into two broad functional themes (see Supporting Information S1), cell-environment interaction (focal adhesion, ECM-receptor interaction, tight junction and cell adhesion molecules) and growth and metabolism (oxidative phosphorylation, and the three disease pathways Alzheimer's, Parkinson's and Huntington's disease). The first group of pathways involved in cell-environment interaction is consistent with the hypothesis that the ability to recognize and respond to extrinsic signals drives differentiation capacity and cell type specificity. The second theme involves growth and metabolism and again highlights the fact that cell phenotypes across the differentiation spectrum are expected to have different metabolic capacities.

**Table 1:** List of significant KEGG pathways identified by *attract* that discriminate between the four cell types (P-value<0.05).

KEGG Pathway ID	KEGG Pathway Name	Adjusted P-values	Number of Detected Genes	Number of Flat Genes (P-value>0.05)
3010	Ribosome	9.2187E-06	91	7
4512	ECM-receptor interaction	7.6171E-04	45	1
0190	Oxidative phosphorylation	1.1467E-03	92	6
4510	Focal adhesion	1.7173E-03	137	2
5016	Huntington's disease	1.7173E-03	127	8
4530	Tight junction	2.7088E-03	86	0
5012	Parkinson's disease	1.5503E-02	90	7
4060	Cytokine-cytokine receptor interaction	2.1785E-02	62	0
4514	Cell adhesion molecules (CAMs)	2.1785E-02	59	1
5010	Alzheimer's disease	3.3719E-02	120	8
4080	Neuroactive ligand-receptor interaction	3.7780E-02	47	1

Flat genes are genes which do not show significant changes across the cell types (P-value>0.05 from a LIMMA model).

Because many genes are annotated to multiple KEGG pathways, so we also examined the overlap between pathways identified by *attract* to determine whether a small number of genes drove the significance of multiple pathways. The top ranked pathway, ribosome, was comprised of genes that did not overlap with any of the other significant pathways. However, there was substantial redundancy in the membership of other significant pathways with the highest pair-wise overlap being 74%. For highly overlapping pathways pairs, smaller pathways with lower representation of common genes (such as ECM, with 45 members) ranked higher than larger with which they overlapped (such as focal adhesion, with 137 members).

**Figure 3:**
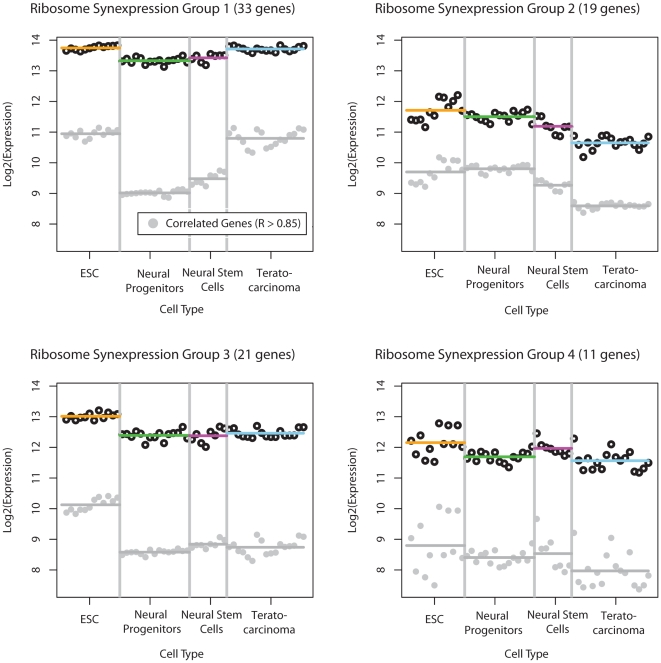
Synexpression groups and their correlated sets for the Ribosome pathway. Log (2) Expression on the x-axis and sample categories are listed across the Y-axis. Each black circle represents the average gene expression for each sample within a group, and corresponding colored bar the average expression for that cell type; Similarly, each grey circle represents the average correlated gene expression for each sample and grey bar the average expression for that cell type.

Despite an overlap in membership, the synexpression groups derived for each pathway were unique—reflecting pathway-specific expression profiles rather than a global pattern driven by a few genes (see Figure 3, Supporting Information S1). We also examined the “flat genes”—those which displayed no significant difference across sample classes—but found a negligible number in most of the pathways ranked as significant.

The synexpression groups that best distinguished ESCs from the other cell types and were seeded from three pathways: ribosome, cell adhesion molecules, and tight junction. The union of these three groups was a large gene set including known pluripotency factors such as Oct4, c-Myc and Nanog. In order to validate the *attract* approach, we assessed the final correlated gene sets in the Ingenuity Pathway Analysis (IPA) platform. The list of genes that make up the PluriNet was downloaded from http://www.openstemcellwiki.org/ and loaded into IPA. The combined ESC-specific synexpression groups were derived by combining the gene lists from the pathways involving the tight junction, ECM-receptor interaction and cell adhesion molecules (CAM) as defined in KEGG. The enrichment of PluriNet genes with this combined ESC-specific group was evaluated in IPA using a Fisher's exact test, and resulted in a one-sided P-value<1.36×10^−39^. The PluriNet originally described by Müller [1] was significantly over represented in this gene set with a particular emphasis on chromatin modifiers such as the DNA methyltransferases (see Supporting Information S1). However, the gene network found using *attract* expanded the signaling context of these chromatin regulators. We observed the convergence of several developmental inputs such as beta-catenin, FGF and IGF receptors and ligands, as well as calcium signaling and cell-cell adhesion proteins that were not seen in PluriNet (Supporting Information S1). Many of the novel components in our network are predicted to be extracellular proteins or present on the ES cell membrane, and several of these include proteoglycans and lectins that had not previously been described in the context of stem cell signaling networks.

### Comparison with pathway annotation of a ranked gene list

The most commonly used approach for the analysis of gene expression data is to use statistical methods to compare gene expression profiles between phenotypic groups to identify “significant” gene sets that are then used in a *post hoc* functional enrichment analysis using a tool such as DAVID [8], [9] to test for an over-representation of particular Gene Ontology classes or KEGG pathways. This approach relies on a “significant gene” list that often represents one aspect of differential gene expression and assumes that all genes in that functional group should behave in the same way. The first step in a*ttract* is a feature selection using a pathway-based significance test using GSEA-ANOVA, so we compared *attract* to this more common selection/enrichment method.

The rationale behind a ranked-gene list annotation approach is to reduce genome-wide data to a subset of informative genes, where rankings are usually based on the degree of difference (p-value or fold change) between the variables being examined. We first used a implementation of LIMMA(version 3.2.3) [10], where we specified a single covariate to represent the different cell types. This used a pair-wise comparison where genes significantly different between any of the 4 cell types were identified, and included a Benjamini-Hochberg correction to produce P-values at three levels: 1×10^−25^ (362 genes), 1×10^−20^ (1127 genes) and 1×10^−15^ (2914 genes). These P-value thresholds were chosen arbitrarily to restrict the size of the resulting gene lists to a size typically used as input for standard enrichment tests.

Enrichment tests are somewhat constrained by the relatively poor representation of annotated genes in mammalian genome. DAVID's EASE statistic uses a modified Fisher's exact test to find pathways whose membership is over-represented among the list of statistically significant genes. GenBank accession numbers were used as the primary identifiers and the whole WG6 version 1 chip was used as the background list. In order to compare the results generated by DAVID (version 6.7) to those of attract, gene sets were restricted to KEGG-defined pathways only. Only the largest ranked-gene list produced any significant pathway hits (P-value<0.05, see Supporting Information S1), but the representation of LIMMA-significant genes in those pathways was rather limited.

DAVID's failure to find meaningful pathways appears to be due to the failure of the initial analysis step to identify meaningful differences between the groups, combined with the shortcomings of the statistical method that underlies DAVID's EASE analysis. This highlights the limitations of the typical ranked gene list approach which clearly missed the most discriminating profiles. A list-based measure simply looks at representation of pathways without considering the expression profiles of individual genes. In contrast, the GSEA-ANOVA test in *attract* takes into the account the ensemble distribution of expression levels represented by a set of genes from the same pathway. Indeed, the pathways identified by *attract* consisted largely of genes that had informative cell-type specific expression and contained few genes that were unchanged across the four cell types (see Table 1 and Figure 3). Therefore, enrichment is assessed by both gene membership and the non-identical contributions from each of these genes as represented by their expression levels in the pathway.

The concept behind *attract* may appear at first glance to be a simple implementation of the ranked gene-list/pathway enrichment approach, but the rationale of identifying discriminating pathways first provides a substantial improvement on the sensitivity and informativeness of the resulting gene sets.

### Comparison with GSEA

GSEA is an alternative pathway-based approach which avoids the ranked-gene list trap, and whose underlying rationale is sympathetic to the first step of *attract.* The original implementation of GSEA [11] allowed for only two-state phenotypic comparisons, but GSEAlm [12], [13] uses a linear model to contrast multiple phenotypic groups. We tested GSEAlm (version 1.6.0) where gene sets were defined by KEGG, with cell type as a single covariate, where we had the choice of using either a model that estimates the absolute effects for each of the four cell types on a gene's expression, or one that estimates the effects of the three cell types relative to a reference type. The former found all 187 KEGG pathways significant at the lowest possible P-value (P-value<1/5000 for 5000 permutations). The latter found 115 pathways that were significant at the 0.05 level (see Supporting Information S1) of which 107 pathways all had the most extreme P-value possible (P<1/5000), making it difficult to identify a subset that captures the cell type-specific differences.

To provide a basis for comparison, we ranked significant pathways from GSEAlm based first on P-value and then on the number of genes they contain. GSEA is known to be influenced by the size of the pathways, and despite corrections for pathway size, this effect that can be clearly seen (Supporting Information S1).

The top-ranked GSEAlm pathway was Pathways in Cancer which is also the largest KEGG pathway, whereas the top-ranked GSEA-ANOVA pathway was Ribosome. These do not share any genes in common, so we tested whether these two pathways provided equivalent discrimination between the four stem cell states. The representation of differentially expressed genes on the two pathways was starkly contrasted: All of the detected genes mapping to the ribosome pathway were differentially expressed between one or more cell type(84/84), with synexpression profiles informative across all 4 cell types; whereas ∼35% (206/569) of the genes on the Pathways in Cancer pathway were differentially expressed, and the syn-expression groups were dominated by differences between the neural progenitors and other cell types.

The biggest difference between GSEAlm and GSEA_ANOVA was the identification and ranking of pathways with significantly different gene members. GSEAlm over-estimated the number of significantly different pathways, and over-represented large pathways. GSEA_ANOVA returned a modest number of differentially expressed pathways, but the differentially expressed genes were highly represented in each of these.

## Discussion

The basis for most expression-based analyses is the search for genes that exhibit patterns of differential expression between phenotypic or experimental groups, followed by meta-analysis to identify potential functional interpretations of the resulting gene lists. This is even true in most approaches focused on identifying co-expression networks from microarray data [14], [15], [16]. This process generally takes the initial significant gene list and reduces it based on a post-hoc application of knowledge about the potential functional roles that the selected genes play. While this is a useful way to annotate large datasets, it often restricts subsequent analyses to well-annotated genes.

On the surface, *attract* represents a subtle shift away from established methods focused on generation of gene lists. These generally pose the question “What genes differentiate the phenotypes?” after which one attempts to place the genes into some biological context through meta-analysis of the identified gene list. Instead, *attract* begins with a systems biology-inspired approach in which we start by asking “What biologically relevant pathways differentiate the phenotype?” This small shift in rationale had a large impact on the number and relevance of the pathways identified. Furthermore, it explicitly grounded the first steps of the pipeline in well-annotated biological processes, which supports hypothesis testing from the earliest stages of the analysis. Having identified candidate pathways, we then decompose pathway-defined gene lists into highly correlated subgroups and extend those by going back to the entire body of data to find additional genes that are highly correlated with each individual subgroup.

The assumption is that these synexpression subgroups are co-regulated is supported, in part, by a *post hoc* functional enrichment analysis which validated that these larger correlated groups are indeed comprised of functionally related genes. The stem cell expression data collected by Müller and colleagues was used to define fifteen stem cell subgroups based on their overall expression phenotype, where individual cell types were able to belong to multiple clusters. For our analysis, we chose four distinct subtypes along the pluripotency spectrum. All of the cell lines were grown in similar growth factor conditions including FGF, EGF, PDGF, and serum. Given the differences between these cell types, one would expect that there should be pathways whose expression patterns distinguish between them.

When using LIMMA and DAVID, we were able to identify individual genes that were differentially expressed across the spectrum of differentiated cells, but only at very low statistical stringency were we able to identify candidate pathways. Applying GSEAlm to the same dataset produced a very unfocused result that lacked discriminating power at the pathway or phenotype level.;the largest and most significant pathway, Pathways of Cancer, had similar expression profiles for human ESCs and the teratocarcinomas, the two most phenotypically diverse cell lines. This became more obvious when we separated this pathway into synexpression groups where three of the four groups clustered the ESCs and teratocarcinomas together (Supporting Information S1).

In contrast, *attract* clearly identified two overarching biological themes—cell growth/metabolism and cell-environment interactions—as the most informative discriminators between the four cell types. This is much more consistent with our understanding of the phenotypes, reflecting not just their array of responses to the extrinsic growth factors, but also their inherent capacity to respond to those signals. The pathways and synexpression groups identified by *attract* provide a model that bridges many aspects of the phenotypes that are unique to ESCs with the chromatin landscape that is associated with pluripotency. Many of the genes implicated in ESC function sit at the interface between cell and extracellular environment and the pathways that they correlate with highlight physical aspects of ESC growth—the characteristic colonies with close interactions between the cells, increased protein synthesis capacity, cell polarity, and the role of physical structures such as the cell cilium.

*attract* also identified the ribosomal pathway as significant, and it is driven by elevated expression across the pathway in ESC. This is consistent with previous reports that ribosomal proteins are elevated in tissues such as ovary, uterus and embryonic stem cells. Ribosome biogenesis is tightly regulated, and has been previously linked to the translational demands of the cell, such that cells that are highly proliferative have higher expression of ribosome genes. For example, increases in ribosomal biogenesis have been associated with proliferative disorders such as cancer. We do not see strong evidence of altered rates of proliferation in human ESC compared to the other cell types included in this comparison. Rather, it is tempting to speculate that ESCs have a higher translational capacity to cope with the demands of differentiation, which requires fundamental shifts in cellular morphology and phenotype.

Understanding the mechanisms that drive cell-fate transitions is one of the greatest challenges in modern biological science. Although there may be many factors influencing these transitions, including both genetic and epigenetic effects, the manifestations of those factors is the expression state of that cell's genes. Stuart Kauffman suggested that cellular states represent particular attractors in the complex adaptive landscape represented by gene expression state space, and our modern interpretation of these attractors is that they represent the activation and coordinated regulation of particular key pathways. The *attract* method builds on that assumption by using a knowledge-driven approach to test the hypothesis that key pathways are important, and then to find other potential components of those pathways or associated regulatory networks.

In our analysis of ESCs, neural stem cells, neural progenitors and teratocarcinomas, we discovered that there are a small number of pathways that are essential to explain the phenotypic differences we observe. Signal transduction pathways that interact with the ECM are functionally important for maintenance of the self-renewal state of ESCs. Pathways relating to key cellular structures, like focal adhesions and the cell adhesion molecules are critical to both ensuring proper attachment of stem cells to their stem cell niches, but also in the upkeep of the stem cell niche so that it is able to retain its stem cells and recruit others when needed. The use of *attract* allowed us to identify those well annotated pathways which best contrasted the cell types of interest, without assuming that all elements of the pathway should behave in an identical manner. Furthermore, it integrates novel elements by virtue of their correlated expression patterns to well annotated functional processes.

Although we used stem cell expression data as a way of demonstrating the power of *attract*, there is no reason to believe that its applicability is limited to this or any other system. Because of the assumptions underlying *attract*, it should be useful in discovering the core networks, pathways, and systems that define cell states.

## Supporting Information

Supporting Information S1 (PDF)
