# Supplementary material for: attract: A Method for Identifying Core Pathways That Define Cellular Phenotypes
Source: PLoS One. 2011 Oct 14;6(10):e25445. doi: 10.1371/journal.pone.0025445 (PMC3194807; doi:10.1371/journal.pone.0025445)
Supplement: Supporting Information S1 — (PDF) [file pone.0025445.s001.pdf]

Mar, JC et al, ***attract*: A method for identifying core pathways that define cellular phenotypes. Supplemental information**

Supplemental Information

Figure S1: Union of the three ESC-related pathways and the PluriNet. Ingenuity IPA visualization of the 3 top ranked attract pathways in the Mueller dataset, and the overlay with the Plurinet gene list originally identified by the Mueller analyses as diagnostic of pluripotency.

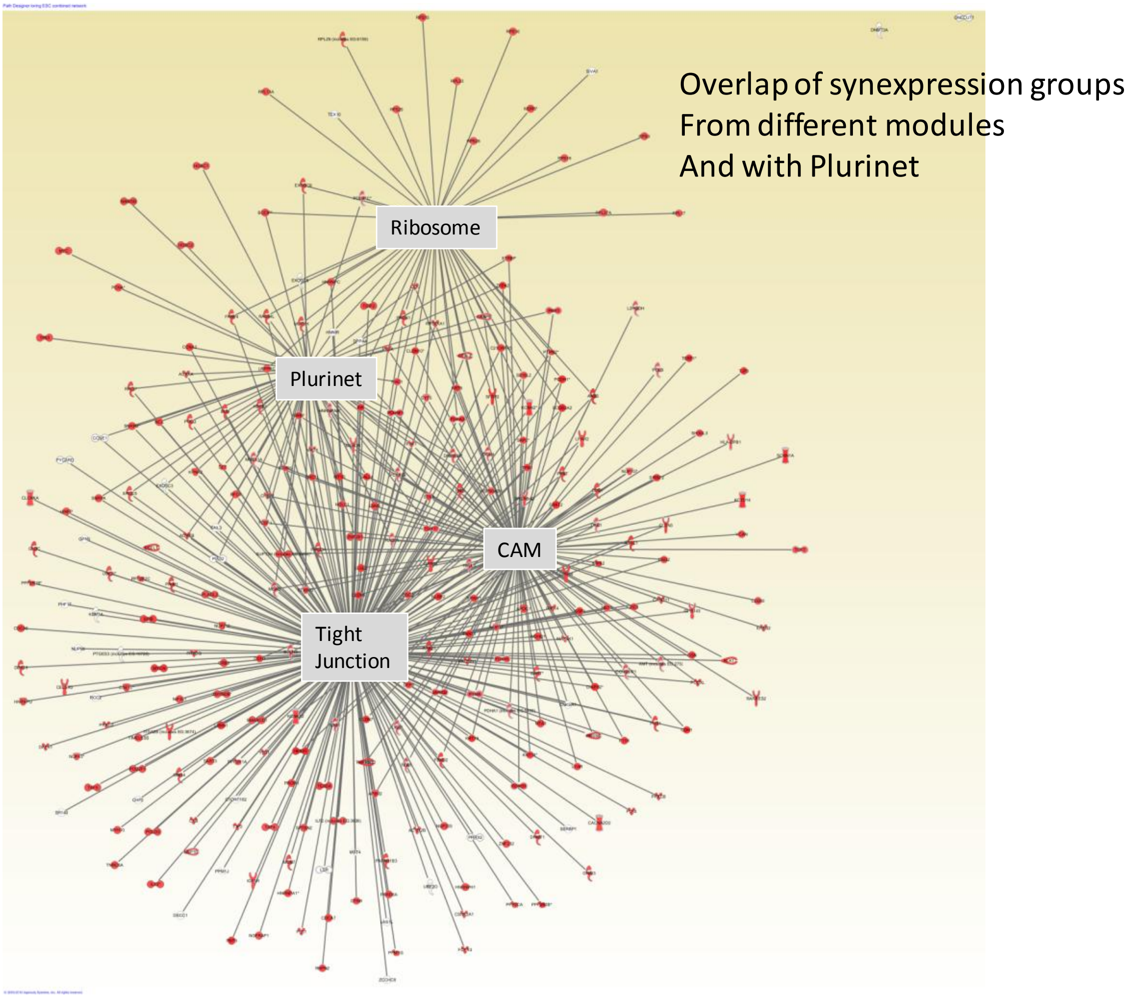

Mar, JC et al, ***attract*: A method for identifying core pathways that define cellular phenotypes. Supplemental information**

Figure S2: Ingenuity network map of the three ESC-related pathways. Plurinet genes are highlighted in green. Ingenuity IPA visualization of the 3 top ranked attract pathways in the Mueller dataset, and the overlay with the Plurinet gene list originally identified by the Mueller analyses as diagnostic of pluripotency. This figure highlights the connectivity between members of the plurinet and attract pathways with literature-annotated edges.

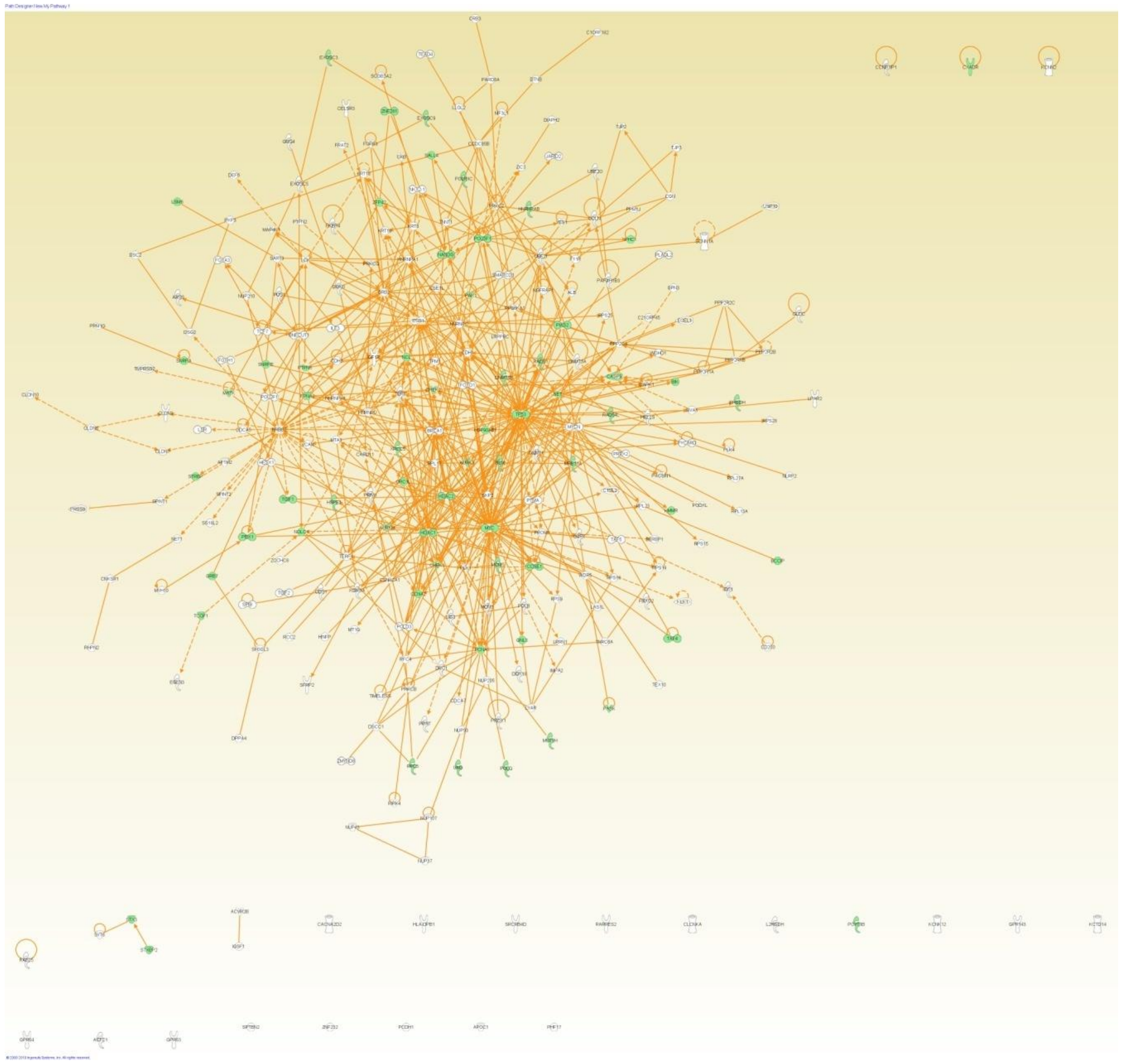

Mar, JC et al, ***attract*: A method for identifying core pathways that define cellular phenotypes. Supplemental information**

Figure S3: Ingenuity IPA visualization of parts of the ESC-related networks cover developmental inputs that feed into the Oct4 and Nanog signaling networks. Plurinet genes are highlighted in green.

Path Designer nanog network

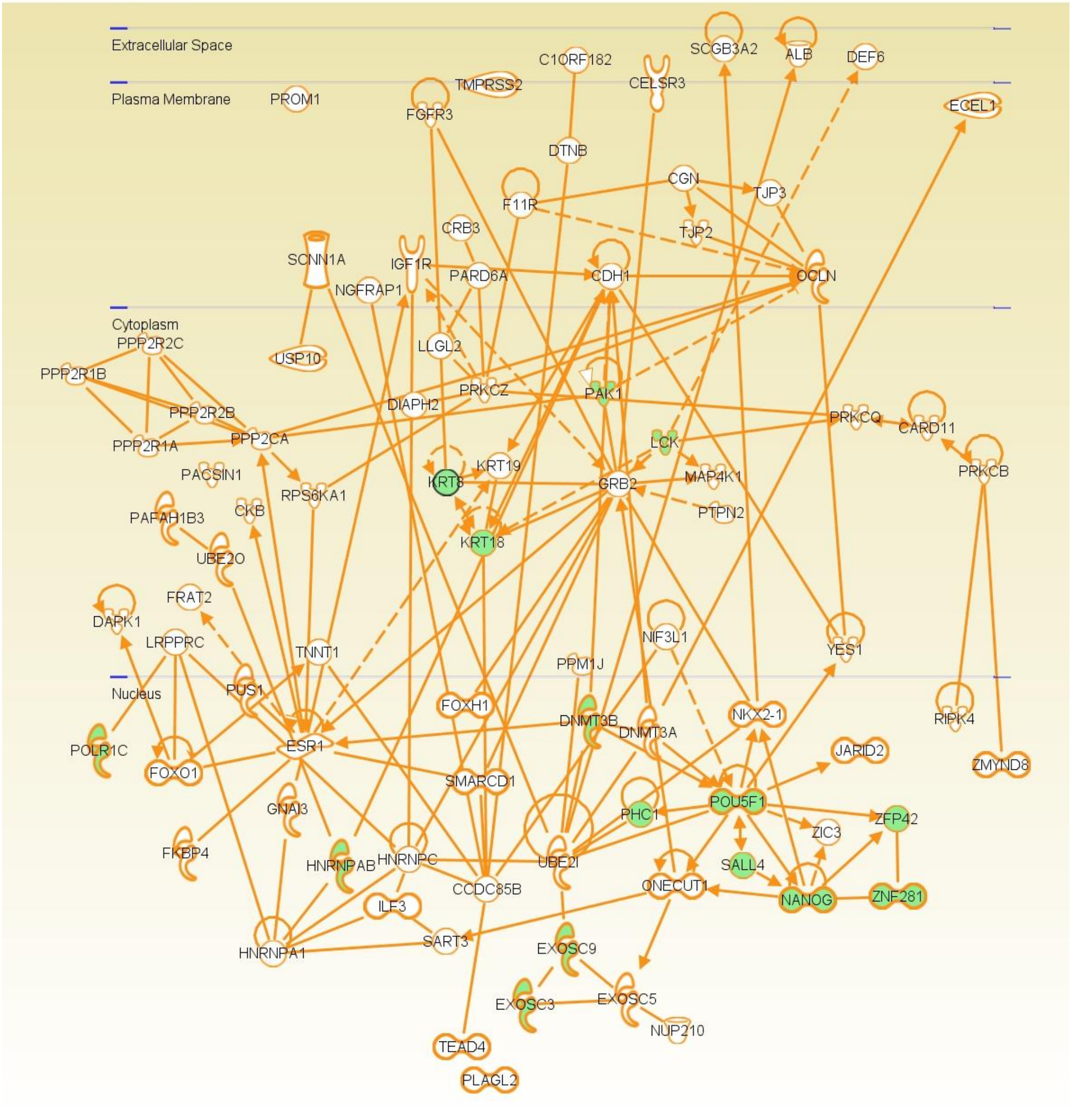

© 2000-2010 Ingenuity Systems, Inc. All rights reserved.

Mar, JC et al, ***attract*: A method for identifying core pathways that define cellular phenotypes. Supplemental information**

Figure S4: Synexpression groups for the ECM pathway identified using attract.

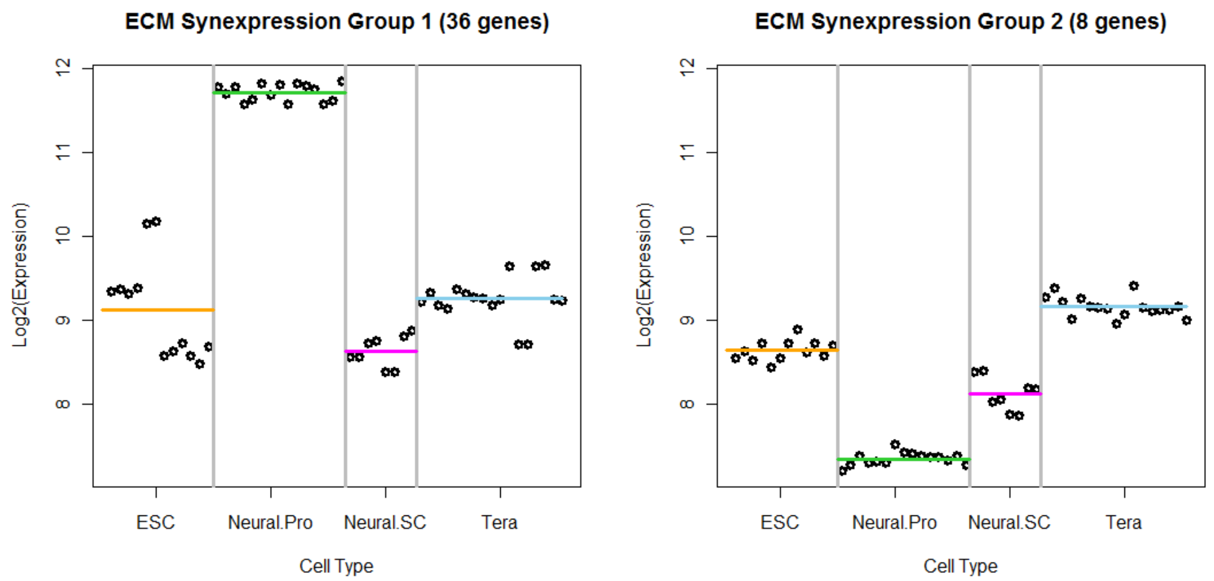

Figure S5: Synexpression groups for the oxidative phosphorylation pathway identified using attract.

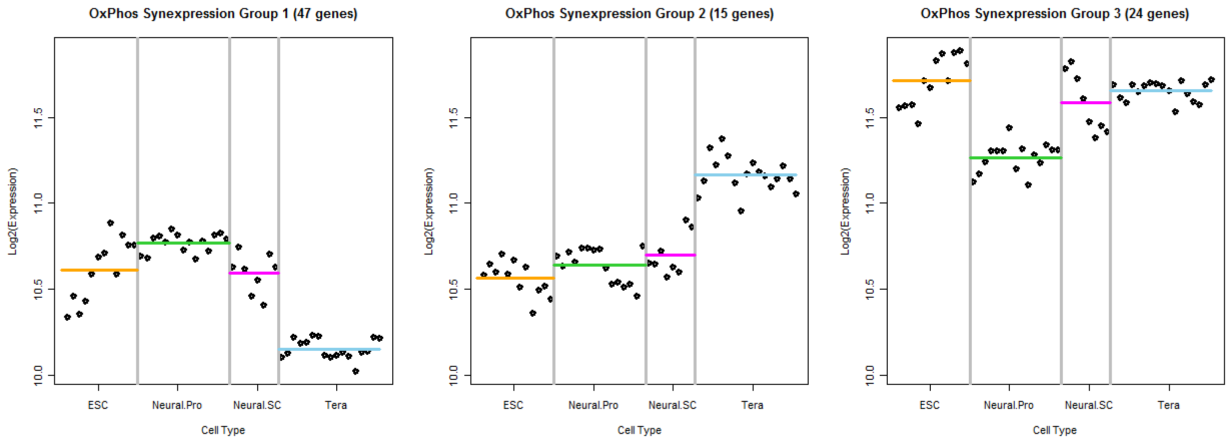

Mar, JC et al, ***attract*: A method for identifying core pathways that define cellular phenotypes. Supplemental information**

Figure S6: Synexpression groups for the genes in Pathways in cancer, identified by GSEAlm as the most significant and most representative pathway.

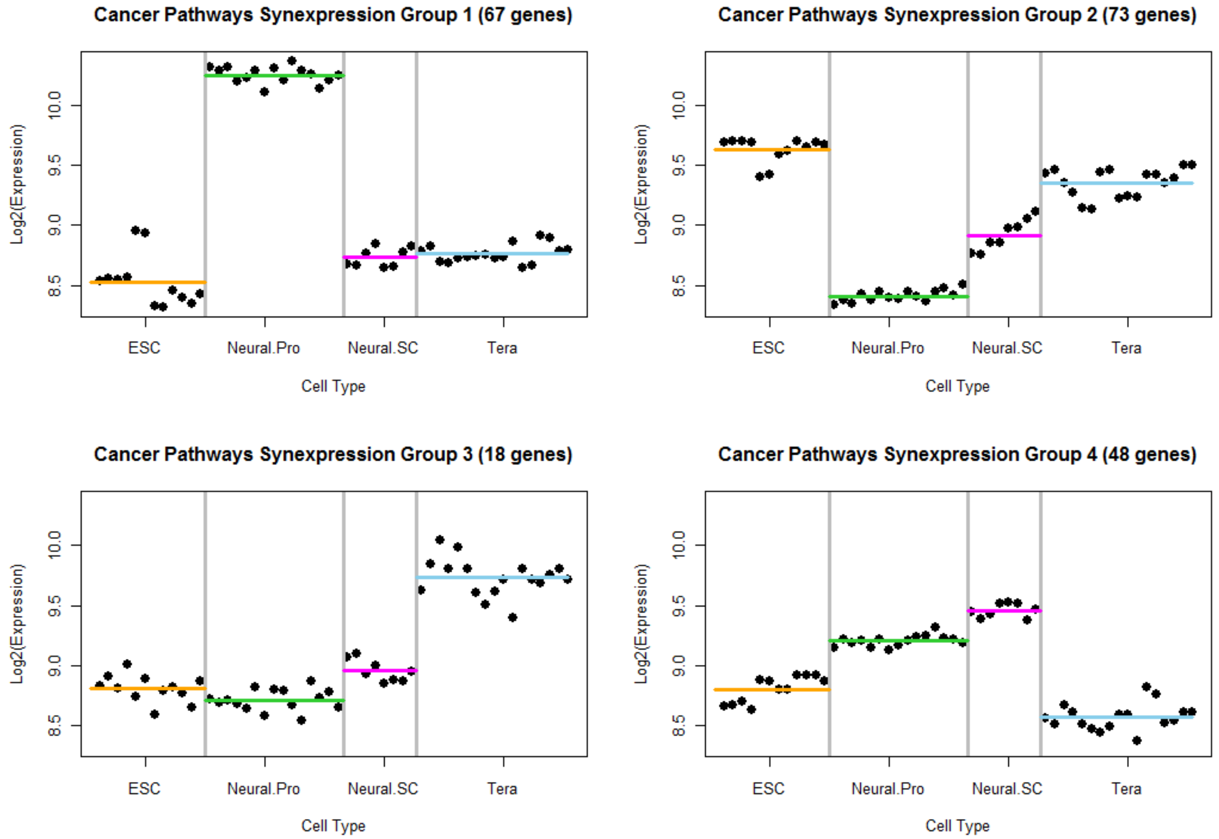

Mar, JC et al, ***attract*: A method for identifying core pathways that define cellular phenotypes. Supplemental information**

Table S1: Overlap between KEGG pathways is as high as 74% in some of the significant pathways identified by attract.

KEGG Pathway ID	03010	4512	0190	4510	5016	4530	5012	4060	4514	5010	4080	KEGG Pathway Name	Adjusted P-values
**03010**	91	0	0	0	0	0	0	0	0	0	0	Ribosome	9.22×10^{-06}
**4512**	0	45	0	37	0	0	0	0	4	0	0	ECM-receptor interaction	7.62×10^{-04}
**0190**	0	0	92	0	63	0	63	0	0	63	0	Oxidative phosphorylation	1.15×10^{-03}
**4510**	0	37	0	137	0	21	0	7	2	8	0	Focal adhesion	1.72×10^{-03}
**5016**	0	0	63	0	127	0	72	0	0	71	1	Huntington's disease	1.72×10^{-03}
**4530**	0	0	0	21	0	86	0	0	12	0	0	Tight junction	2.71×10^{-03}
**5012**	0	0	63	0	72	0	90	0	0	67	0	Parkinson's disease	1.55×10^{-02}
**4060**	0	0	0	7	0	0	0	62	0	1	0	Cytokine-cytokine receptor interaction	2.18×10^{-02}
**4514**	0	4	0	2	0	12	0	0	59	0	0	Cell adhesion molecules (CAM)	2.18×10^{-02}
**5010**	0	0	63	8	71	0	67	1	0	120	1	Alzheimer's disease	3.37×10^{-02}
**4080**	0	0	0	0	1	0	0	0	0	1	47	Neuroactive ligand-receptor interaction	3.78×10^{-02}

Table S2: Ranked lists of over-represented KEGG pathways using DAVID for generated by applying a P-value cut-off of 1×10^{-15}, where the top twenty pathways are shown. The P-values were adjusted to control the false discovery rate, using the Benjamini-Hochberg correction method. Significant pathways (P-value < 0.05) have been colored orange in the table.

ID	Pathway Name	Adjusted P-values
04510	Focal adhesion	0.0016
05222	Small cell lung cancer	0.0293
04512	ECM-receptor interaction	0.0357
04530	Tight junction	0.0828
03030	DNA replication	0.3784
05221	Acute myeloid leukemia	0.3788
04142	Lysosome	0.3797
03040	Spliceosome	0.3499
05200	Pathways in cancer	0.3467
03410	Base excision repair	0.3244
05216	Thyroid cancer	0.3053
03018	RNA degradation	0.3964
05412	Arrhythmogenic right ventricular cardiomyopathy (ARVC)	0.4258
05212	Pancreatic cancer	0.4258
05219	Bladder cancer	0.5099
00230	Purine metabolism	0.5410
04514	Cell adhesion molecules (CAMs)	0.7121
05213	Endometrial cancer	0.7237
05215	Prostate cancer	0.7194
04670	Leukocyte transendothelial migration	0.7194

Table S3: Ranked lists of over-represented KEGG pathways using DAVID for generated by applying a P-value cut-off of 1×10^{-20}, where the top twenty pathways are shown. The P-values were adjusted to control the false discovery rate, using the Benjamini-Hochberg correction method. Significant pathways (P-value < 0.05) have been colored orange in the table.

ID	Pathway Name	Adjusted P-values
04510	Focal adhesion	0.3709
04530	Tight junction	0.2950
04142	Lysosome	0.4069
04512	ECM-receptor interaction	0.4593
04514	Cell adhesion molecules (CAMs)	0.5522
05214	Glioma	0.8130
05219	Bladder cancer	0.7916
00450	Selenoamino acid metabolism	0.8122
05416	Viral myocarditis	0.7816
05222	Small cell lung cancer	0.8438
04810	Regulation of actin cytoskeleton	0.8373
05200	Pathways in cancer	0.8116
04722	Neurotrophin signaling pathway	0.7961
05215	Prostate cancer	0.8294
05213	Endometrial cancer	0.8169
04670	Leukocyte transendothelial migration	0.8109
00330	Arginine and proline metabolism	0.7996
03410	Base excision repair	0.8512
04012	ErbB signaling pathway	0.8791
00480	Glutathione metabolism	0.8685

Table S4: Ranked lists of over-represented KEGG pathways using DAVID for generated by applying a P-value cut-off of 1×10^{-25}, where the top twenty pathways are shown. The P-values were adjusted to control the false discovery rate, using the Benjamini-Hochberg correction method. Significant pathways (P-value < 0.05) have been colored orange in the table.

ID	Pathway Name	Adjusted P-values
05219	Bladder cancer	0.2647
04530	Tight junction	0.3611
05200	Pathways in cancer	0.7539
04514	Cell adhesion molecules (CAMs)	0.8849
04510	Focal adhesion	0.8497
04672	Intestinal immune network for IgA production	0.8833
00450	Selenoamino acid metabolism	0.8471
05130	Pathogenic Escherichia coli infection	0.8438
04142	Lysosome	0.8128
04540	Gap junction	0.8250
00460	Cyanoamino acid metabolism	0.8279
05416	Viral myocarditis	0.8827
00430	Taurine and hypotaurine metabolism	0.8809
04512	ECM-receptor interaction	0.9240
04670	Leukocyte transendothelial migration	0.9116
05410	Hypertrophic cardiomyopathy (HCM)	0.9190
00480	Glutathione metabolism	0.9134
05215	Prostate cancer	0.9255
05414	Dilated cardiomyopathy	0.9195
05213	Endometrial cancer	0.9138

Mar, JC et al, ***attract*: A method for identifying core pathways that define cellular phenotypes. Supplemental information**

Table S5: Degree of overlap between the lists of significantly differentially expressed genes generated from LIMMA (as inputs to DAVID) and the pathways identified by GSEA-ANOVA from *attract*. Two of the significantly enriched pathways that were identified by DAVID are highlighted in yellow; these two pathways demonstrate a higher percentage of overlap than most of the other pathways.

KEGG Pathway Name	Number of Detected Genes	Overlap (%) with List1 [P-value $< 1\times10^{-15}$]	Significance of Overlap (P-value)	Overlap (%) with List2 [P-value $< 1\times10^{-20}$]	Significance of Overlap (P-value)	Overlap (%) with List3 [P-value $< 1\times10^{-25}$]	Significance of Overlap (P-value)
Ribosome	91	8	0.999	2	0.993	0	0.949
ECM-receptor interaction	45	27	2.08×10^{-09}	12	0.000154	4	0.0137
Oxidative phosphorylation	92	10	0.992	3	0.977	1	0.798
Focal adhesion	137	63	1.31×10^{-11}	29	7.37×10^{-06}	9	0.0128
Huntington's disease	127	19	0.947	4	0.994	1	0.918
Tight junction	86	39	$9.69\times10\text{-}^{08}$	21	8.56×10^{-06}	9	0.000400
Parkinson's disease	90	11	0.978	5	0.856	2	0.553
Cytokine-cytokine receptor interaction	62	28	4.68×10^{-06}	11	0.0108	5	0.0138
Cell adhesion molecules (CAMs)	59	32	3.82×10^{-09}	18	8.03×10^{-07}	7	0.000529
Alzheimer's disease	120	24	0.557	8	0.801	2	0.745
Neuroactive ligand-receptor interaction	47	20	0.000211	12	0.000249	5	0.00357
Total List Size		2914		1127		362	

Table S6: Ranked list of pathways identified by GSEAlm where pathways reflect those that have the most significant changes between the ESC group and the neural progenitor cell lines. Pathway list is ranked first by the P-value and second by the number of detected genes annotated to the pathway and only the top twenty results are shown.

KEGG Pathway ID	KEGG Pathway Name	P-value	Number of Detected Genes
5200	Pathways in cancer	0.00019996	212
4010	MAPK signaling pathway	0.00019996	157
4810	Regulation of actin cytoskeleton	0.00019996	139
4510	Focal adhesion	0.00019996	137
4144	Endocytosis	0.00019996	126
4142	Lysosome	0.00019996	97
4910	Insulin signaling pathway	0.00019996	97
4062	Chemokine signaling pathway	0.00019996	95
4722	Neurotrophin signaling pathway	0.00019996	95
4360	Axon guidance	0.00019996	95
4666	Fc gamma R-mediated phagocytosis	0.00019996	71
4020	Calcium signaling pathway	0.00019996	68
4660	T cell receptor signaling pathway	0.00019996	66
4270	Vascular smooth muscle contraction	0.00019996	66
4670	Leukocyte transendothelial migration	0.00019996	65
4912	GnRH signaling pathway	0.00019996	64
5210	Colorectal cancer	0.00019996	63
4012	ErbB signaling pathway	0.00019996	63
5220	Chronic myeloid leukemia	0.00019996	62
4350	TGF-beta signaling pathway	0.00019996	62

Table S7: List of pathways from GSEAlm analysis ranked by pathway size. The dominance of most significant pathways is apparent in the top twenty pathways. Pathways with the most extreme significant P-value have been highlighted in yellow.

KEGG Pathway ID	KEGG Pathway Name	P-value	Number of Detected Genes
1100	Metabolic pathways	0.986402719	692
5200	Pathways in cancer	0.00019996	212
4010	MAPK signaling pathway	0.00019996	157
4810	Regulation of actin cytoskeleton	0.00019996	139
4510	Focal adhesion	0.00019996	137
5016	Huntington's disease	1	127
4144	Endocytosis	0.00019996	126
5010	Alzheimer's disease	0.00959808	120
3040	Spliceosome	1	111
4110	Cell cycle	1	110
4120	Ubiquitin mediated proteolysis	1	108
230	Purine metabolism	1	107
4310	Wnt signaling pathway	0.936612677	106
4142	Lysosome	0.00019996	97
4910	Insulin signaling pathway	0.00019996	97
4062	Chemokine signaling pathway	0.00019996	95
4722	Neurotrophin signaling pathway	0.00019996	95
4360	Axon guidance	0.00019996	95
4114	Oocyte meiosis	1	93
190	Oxidative phosphorylation	0.99480104	92
